# Characterisation of five candidate genes within the ETEC F4ab/ac candidate region in pigs

**DOI:** 10.1186/1756-0500-4-225

**Published:** 2011-06-30

**Authors:** Mette Jacobsen, Susanna Cirera, David Joller, Gloria Esteso, Steffen S Kracht, Inger Edfors, Christian Bendixen, Alan L Archibald, Peter Vogeli, Stefan Neuenschwander, Hans U Bertschinger, Antonio Rampoldi, Leif Andersson, Merete Fredholm, Claus B Jørgensen

**Affiliations:** 1 Department of Basic Animal and Veterinary Sciences, University of Copenhagen, 1870 Frederiksberg, Denmark; 2 Institute of Agricultural Sciences, ETH Zurich, 8092 Zurich, Switzerland; 3 Genomica y Mejora Animal, Departamento de Genética, Universidad de Córdoba, Campus de Rabanales, 14071, Córdoba, Spain; 4 School of Natural Sciences, Linnaeus University, 391 82 Kalmar, Sweden; 5 Department of Genetics and Biotechnology, Aarhus University, 8830 Tjele, Denmark; 6 Division of Genetics and Genomics, The Roslin Institute and R(D)SVS, University of Edinburgh, Roslin, Midlothian EH25 9PS, UK; 7 Department of Medical Biochemistry and Microbiology, Uppsala University, 751 23 Uppsala, Sweden

## Abstract

**Background:**

Enterotoxigenic *Escherichia coli *(ETEC) that express the F4ab and F4ac fimbriae is a major contributor to diarrhoea outbreaks in the pig breeding industry, infecting both newborn and weaned piglets. Some pigs are resistant to this infection, and susceptibility is inherited as a simple dominant Mendelian trait. Indentifying the genetics behind this trait will greatly benefit pig welfare as well as the pig breeding industry by providing an opportunity to select against genetically susceptible animals, thereby reducing the number of diarrhoea outbreaks. The trait has recently been mapped by haplotype sharing to a 2.5 Mb region on pig chromosome 13, a region containing 18 annotated genes.

**Findings:**

The coding regions of five candidate genes for susceptibility to ETEC F4ab/ac infection (*TFRC, ACK1, MUC20, MUC4 *and *KIAA0226*), all located in the 2.5 Mb region, were investigated for the presence of possible causative mutations. A total of 34 polymorphisms were identified in either coding regions or their flanking introns. The genotyping data for two of those were found to perfectly match the genotypes at the ETEC F4ab/ac locus, a G to C polymorphism in intron 11 of *TFRC *and a C to T silent polymorphism in exon 22 of *KIAA0226*. Transcriptional profiles of the five genes were investigated in a porcine tissue panel including various intestinal tissues. All five genes were expressed in intestinal tissues at different levels but none of the genes were found differentially expressed between ETEC F4ab/ac resistant and ETEC F4ab/ac susceptible animals in any of the tested tissues.

**Conclusions:**

None of the identified polymorphisms are obvious causative mutations for ETEC F4ab/ac susceptibility, as they have no impact on the level of the overall mRNA expression nor predicted to influence the composition of the amino acids composition. However, we cannot exclude that the five tested genes are *bona fide *candidate genes for susceptibility to ETEC F4ab/ac infection since the identified polymorphism might affect the translational apparatus, alternative splice forms may exist and post translational mechanisms might contribute to disease susceptibility.

## Background

Diarrhoea in neonatal and newly weaned pigs is a serious welfare problem and a financial burden in pig production. Some piglets are resistant to certain types of bacterial infection and identifying the genetic cause for susceptibility is a prerequisite for an effective selection program. One of the bacteria causing diarrhoea is enterotoxigenic *Escherichia coli *(ETEC) that expresses the F4 fimbriae with the variants F4ab, F4ac and F4ad. These fimbriae adhere particularly to specific receptors on the brush borders of the enterocytes of the small intestine [1]. The enterocytes of pigs susceptible to ETEC F4ab and F4ac diarrhoea express a receptor that interacts with F4ab and F4ac adhesins, and the expression of this receptor is inherited as an autosomal dominant Mendelian trait [2]. We therefore expect that the candidate region for susceptibility to ETEC F4ab/ac infection contains a gene encoding the receptor itself, an endogenous ligand or a product which modifies the structure and function of the receptor.

The locus responsible for susceptibility has been mapped to pig chromosome 13 in the q41 region by two independent linkage studies [3,4] and the candidate region has subsequently been narrowed down to 5.7 cM [5]. Recently, the locus has been further refined by haplotype sharing to a region of 3.1 Mb [6]. This region corresponds to approximately 2.5 Mb in the published pig map in Ensembl (Sscrofa9), from position 100.680.954 to 103.192.935, and contains 18 annotated porcine genes.

Protein-protein interaction studies have revealed that the F4ab/ac receptor, which interacts with the F4ab/ac fimbriae, is most likely to be either a mucin-like sialoglycoprotein or a transferrin-like protein [7-9]. Previously, intron 7 of the mucin 4 gene (*MUC4*) was shown to contain a non-causative polymorphism in complete LD with susceptibility to ETEC infection [10]. Five candidate genes in the 2.5 Mb region including and in the vicinity of *MUC4 *were therefore screened for mutations in the coding sequences and in parts of the intervening introns in order to identify possible genetic alterations in ETEC F4ab/ac susceptible animals. In addition to *MUC4 *these genes were the transferrin receptor (*TFRC)*, the tyrosine kinase, non-receptor, 2 *(ACK1)*, the mucin 20 (*MUC20) *and the *KIAA0226 *genes. Both *MUC4 *and *MUC20 *are striking candidate genes for susceptibility, as both are membrane bound, highly glycosylated proteins, and contribute to the immune defence system by being major components of the glycocalyx layer of epithelial cells. In addition, they are both highly abundant in the gastrointestinal tract [11,12]. TFRC is essential for the transport of iron from the transferrin protein into the cell, and *E. coli *bacteria are dependent on iron availability for survival and propagation. The cDNA sequence for *TFRC *has previously been explored for mutations associated with ETEC F4ab/ac susceptibility [13,14], but none of these studies explored the exon-intron junctions for mutations that could interfere with proper splicing of the transcript. ACK1 has been shown to impact the expression level of *TFRC *[15], thereby making *ACK1 *a potential candidate gene. Little is known about the protein encoded by *KIAA0226*, called Rundataxin due to the presence of a RUN element in the primary sequence and its association with the human disease Ataxia [16].

Since animals susceptible to ETEC infection express the F4ab/ac receptor irrespective of the presence of bacteria and since susceptibility might be reflected at the level of gene transcription, we investigated the transcriptional profile by quantitative real time PCR (qPCR) of the five candidate genes in 11 different tissues and intestinal cells from both ETEC F4ab/ac susceptible and resistant animals. The research hypothesis behind selection of the five invested candidate genes for ETEC F4ab/ac susceptibility was two-fold: 1) Their location in a region closely linked to susceptibility and 2) the ontology of the encoded proteins.

## Methods

### Animals

Investigation of the genetic variations in the five candidate genes, *TFRC, ACK1, MUC4, MUC20 *and *KIAA0226*, was performed on genomic DNA from two resistant Wild Boars and two homozygous susceptible Large White sows. All four animals were phenotyped for both F4ab and F4ac adhesion [1] and have previously been described [6].

Additional genotyping of the six identified polymorphisms were performed on genomic DNA from a total of 42 pigs; six susceptible Large White sows, two susceptible and four resistant Yorkshire pigs, one susceptible Landrace and one resistant Landrace pig, 26 susceptible and two resistant crossbreeds between Duroc, Landrace and Yorkshire. They were all phenotyped for both F4ab and F4ac adhesion as previously described [1,4]

For the expression profiles, four 3-months old siblings were slaughtered (2 female and 2 male crossbreeds between Landrace, Yorkshire and Duroc), and 11 tissues (lung, liver, kidney, lymph nodes, muscle, *pancreas, cerebral cortex, colon, jejunum, duodenum *and *ileum*) were collected, immediately frozen in liquid nitrogen and stored at -80°C until RNA purification. No information on F4ab/ac status was available from these animals.

Furthermore, expression profiles of the candidate genes in enterocytic cells from *jejunum *were investigated in 5 resistant and 5 susceptible Yorkshire animals that were phenotyped for both F4ab and F4ac adhesion as previously described [1].

All samples were collected from animals kept under conditions required for farm animals in Denmark. No approvals from ethics committees were required for this study.

### Extraction of DNA and RNA

DNA was extracted from whole blood using the Epicenter kit (Promega) according to the manufacturer's protocol. The amount and quality of DNA was measured using Nanodrop 1000 (Thermo Fisher Scientific). Total RNA was extracted from tissue samples (two biological replicates per tissue and animal) using TriReagent^® ^(Molecular Research Centre), according to the manufacturer's recommendations. After purification the RNA was DNase treated using spin-column purification (Qiagen). Total RNA was quantified by optical density and the quality was evaluated by ribosomal RNA 28S/18S band inspection by gel electrophoresis and by RNA integrity number (RIN, 2100 Bioanalyzer, Agilent technologies). A RIN number of five was chosen as minimum threshold for acceptance.

### Primer design, PCR and sequencing

Using mRNA sequences from either human and/or pig of the five candidate genes *TFRC *[GenBank: NM_214001], *ACK1 *[GenBank: NM_001010938 and GenBank: NM_005781], *MUC4 *[GenBank: NM_018406], *MUC20 *[GenBank: NM_001113440] and *KIAA0226 *[GenBank: NM_001145642 and NM_014687] to blast against the porcine sequenced BAC-clones [GenBank: CU695181, CU914410, CU468995, CU694544 and FP102930], it was possible to identify all the genomic coding sequences in the pig, except for *TFRC *exon 1, *ACK1 *exon 1, *MUC4 *exon 2 and *KIAA0226 *exon 1. Primers were designed in intron sequences flanking the exons using the PRIMER3 software http://frodo.wi.mit.edu. For a complete list of primer sequences, amplicon sizes, and coverage, see additional file 1. The primer pairs were all tested for amplification efficiency over a range of annealing temperatures using TEMPase Hot Start DNA polymerase and amplicons were amplified according to the manufacturer's recommendations (Amplicon). 5 μl of the PCR products were visualised on a 1 % agarose gel and the remaining PCR products were purified using Montage PCR_96 _Cleanup kit (Millipore), and sequenced using BigDye Terminator Cycle Sequencing Kit (Applied Biosystems) according to the manufacturer's recommendations. Sequenced products were purified with Montage SEQ_96 _Cleanup kit (Millipore), and resolved on an ABI3130 × l Genetic Analyzer (Applied Biosystems). Traces were assembled and visualised using the LaserGene software V.7.2 (DNASTAR).

### 5' and 3' RACE

To obtain reliable sequence data of the 5' and 3'ends of porcine *MUC4 *and the 5'end of porcine *ACK1*, the SMART™ RACE cDNA amplification kit from Clontech was used. Three primers with a melting temperature of approximately 70°C were designed in exon 3 of *ACK1 *and in exons 1 and 24 of *MUC4 *[Additional file 1], and used in the second strand cDNA synthesis according to the manufacturer's protocol (Clontech). It was possible to amplify three fragments of approximately 400 bp (*ACK1 *5'UTR), 800 bp (*MUC4 *3'UTR) and 250 bp (*MUC4 *5'UTR), respectively. Fragments were purified using a gel purification kit (Qiagen) according to the manufacturer's recommendations. The purified amplicons were then ligated into pGEM^®^-T Easy Vectors (Promega) and cloned into JM109 High Efficiency Competent Cells (Promega). M13 forward and M13 reverse primers were used in a sequencing reaction similar to that described above.

### Analysis of identified polymorphisms

The individual polymorphisms were tested for association with the ETEC F4ab/ac adhesion phenotypes using a chi-squared test, and a standardised linkage disequilibrium value (D') were calculated using Haploview 4.1 [17]. Prediction of splice defects was performed in Human Splice finder - Version 2.4.1 http://www.umd.be/HSF/[18], where the genomic regions of the *TFRC *and *KIAA0226 *genes containing the two polymorphisms in LD with susceptibility to infection (numbers 3 and 34) were exposed to mutation analysis using default settings.

### Real time quantitative RT-PCR (qPCR)

One μg of each total RNA sample was reverse transcribed at 42°C using a mixture of Oligo(dT) and random hexamers (1:3) and Improm-II™ reverse transcriptase (Promega) according to the manual. All primers were designed using PRIMER3 software. Two μl of each cDNA sample (diluted 1:8) was then added to the PCR mixture, consisting of 5 μl QuantiFast™ SYBR Green master mix (Qiagen) and 0.5 mM of each primer (See Table 1 for primer list). All reactions were performed in an Mx3000P™ machine (Stratagene). Thermal cycling conditions were 95°C for 5 min, followed by 40 cycles at 95°C for 15 s and 60°C for 30 s. A dissociation curve was made (95°C for 1 min, 55°C for 30 s, and 95°C for 30 s) to confirm the specificity of the primers.

**Table 1:** Identified polymorphisms in porcine *TFRC, ACK1, MUC4, MUC20 *and *KIAA0226 *genes

Gene	No.	Base	Consequence
***TFRC ***(100.8 Mb)	1	G→A (E4)	Ala→Thr
	2	T→C (E10)	Silent
	3	**G→T (I11)**	**10 bp before branch point**

***ACK1 ***(100.9 Mb)	4	C→G (E1)	Pro→Ala
	5	T→C (E2)	Silent
	6	G→T (E2)	Silent
	7	C→T (E3)	Silent
	8	**A→C (E7)**	**Silent**
	9	C→T (E7)	Silent
	10	T→C (E9)	Silent
	11	C→T (E9)	Silent
	12	**G→A (E10)**	**Silent**
	13	C→A (E12)	Pro→His
	14	A→G (E12)	Silent
	15	G→A (E12)	Silent
	16	G→A (E12)	Val→Met
	17	**A→G (E12)**	**Silent**
	18	G→A (E12)	Arg→His
	19	G→C (E12)	Ser→Thr
	20	C→G (I14)	5 bases before exon 15

***MUC4 ***(101.0 Mb)	21	C→A (E12)	Silent
	22	C→T (I13)	4 bases before exon 14
	23	C→T (I13)	16 bases before exon 14
	24	C→T (I13)	28 bases before exon 14
	25	C→T (E19)	Silent
	26	G→A (E24)	Gly→Ser
	27	T→C (E24)	Silent
	28	G→A (3UTR)	187 bases after TGA
	29	G→A (3UTR)	259 bases after TGA

***MUC20 ***(101.1 Mb)	30	A→C (3UTR)	201 bases after TGA
	31	**C→T (I2)**	**58 bases after exon 2**
	32	C→T (E2)	Silent
	33	A→T (E1)	Silent

***KIAA0226 ***(101.1 Mb)	34	**C→T (E20)**	**Silent**

A total of 34 polymorphisms were identified in the four tested animals (2 resistant Wild Boars and 2 homozygous susceptible Large White sows). Gene names are shown in the first column (gene positions at chromosome 13 in Sscrofa 9 are in brackets), polymorphisms in the second column and E and I in brackets indicate exonic (E) or intronic (I) location of the polymorphisms. The third column shows the nature of the polymorphisms. The six polymorphisms matching the ETEC F4ab/ac genotype perfectly in the four animals are in bold, and the two polymorphisms underlined are also closely associated with the ETEC F4ab/ac status in the additional 42 animals.

Expression levels of the five candidate genes were normalised across tissues using three stable reference genes *TBP, HMBS *and *RPL4 *[19]. The GeNorm software [20] was used to calculate a normalisation factor (NF). Samples were normalised by the NF and fold changes were calculated. Subsequently, the normalised fold changes were tested for normal distribution within each tissue using Instat 3.0 (GraphPad). One factor Analysis of Variance (ANOVA) statistical test was performed to test for significant differences.

## Results

### Porcine orthologues of *TFRC, ACK1, MUC4, MUC20 *and *KIAA0226*

The coding parts of each of the five genes were sequenced and data submitted to GenBank with the following accession numbers; *TFRC *[GenBank: HM070995], *ACK1 *[GenBank: HM070993], *MUC4 *[GenBank: GU983681], *MUC20 *[GenBank: HM070996] and *KIAA0226 *[GenBank: HM849042]. All exons contain consensus donor/acceptor splice sites, and are almost identical in size to their human orthologues, except for the porcine *MUC20 *gene whose exon 2 is orthologues to human exons 2 and 3 combined. However, exon 2 of porcine *ACK1 *differs significantly from human *ACK1 *exon 2 by encoding only 21 amino acids as opposed to 57 amino acids in human.

All exons, except for *TFRC *exon 1, *ACK1 *exon 1, *KIAA0226 *exon 1, and exon 2 of *MUC4*, were investigated for the presence of mutations. The coding part of human *TFRC *begins in exon 2, and due to the sequence diversity in the 5'UTR between human and pig, it was not possible to accurately predict or amplify *TFRC *exon 1 in the pig genome. The same was true for *ACK1 *exon 1, and this exon was therefore not identified in the porcine genome either. Human *KIAA0226 *contains two distinct splice variants [GenBank: NM_014687 and NM_001145642], of which splice variant 1 represents the longest transcript where the first exon is part of the 5'UTR. Due to the sequence diversity in this region, it was not possible to identify exon 1 of *KIAA0226 *by similarity search.

Exon 2 of *MUC4 *contains a 456 base pair sequence, which has been shown to be repeated between 13-21 times in eight pigs from four different breeds (Landrace, Yorkshire, Hampshire and Duroc) according to a Southern blot made by our group (data not shown). This region of the *MUC4 *gene is therefore too large (averaging 8 kb) and complex and thus not possible to analyse using capillary sequencing on PCR product. As *MUC4 *is an obvious prime candidate gene for susceptibility to infection by ETEC F4ab/ac, we identified the whole 5' and 3' untranslated regions using Rapid Amplification of cDNA Ends (RACE).

A total of 34 polymorphisms were identified in the 5 tested genes in the four animals, two F4ab/ac resistant Wild Boars and two F4ab/ac homozygous susceptible Large White sows. 25 polymorphisms were present in the coding regions and nine in the intron sequences (Table 1). All polymorphisms have been annotated in the submitted sequences in GenBank.

Seven of the 25 coding polymorphisms are predicted to result in amino acids changes (Table 1), but none of them can be exclusively associated with the F4ab/ac phenotype in the four tested animals. However, the genotypes at six polymorphic sites matched perfectly the ETEC F4ab/ac phenotypes in these animals (Table 1, bold). These six SNPs were subsequently genotyped in 42 additional and phenotyped animals, and very tight association with the susceptible F4ab/ac phenotype was observed for two of the SNPs; polymorphisms number 3 and 34 (Table 1, bold).

### Transcription profiling

To investigate the expression profiles of the five candidate genes, real-time qPCR was performed using total RNA from 11 different tissues. These tissues originate from four piglets of crosses between the Landrace, the Yorkshire and the Duroc breeds. No information of their ETEC F4ab/ac susceptibility status is known. The expression profiles of the five genes were normalised to three internal controls; *TBP, HMBS *and *RPL4 *[19]. All five genes were expressed in all tissues examined, but at markedly different levels (Table 2). *TFRC *has the highest expression in muscle, and is expressed in all four intestinal tissues at a relatively high level. *ACK1 *has a significantly higher expression level in *cerebral cortex *than in other tissues (P < 0.001), but is still expressed at moderate levels in the four intestinal tissues. *MUC4 *is predominantly expressed in *colon *(P < 0.0001), but present at a low level in all other tissues. *MUC20 *has the highest expression in *duodenum *(P < 0.05) and kidney (P < 0.05), but is also expressed in the three other intestinal tissues at a relatively high level. *KIAA0226 *shows an expression profile quite similar to the expression profile of *ACK1*, with the highest expression in *cerebral cortex*, and moderate expression in all other tissues.

**Table 2:** Expression profile of the porcine *TFRC, ACK1, MUC4, MUC20 *and *KIAA0226 *gene

	*TFRC*	*ACK1*	*MUC4*	*MUC20*	*KIAA0226*
*Cerebral cortex*	68.1	100.0	0.2	3.1	100.0
***Colon***	**51.9**	**17.1**	**100.0**	**22.1**	**26.5**
***Duodenum***	**67.2**	**6.9**	**8.2**	**100.0**	**25.2**
***Ileum***	**29.3**	**10.8**	**4.5**	**38.3**	**39.9**
***Jejunum***	**36.6**	**7.5**	**1.0**	**16.6**	**30.8**
Kidney	56.1	11.3	1.1	71.6	32.7
Liver	62.1	50.4	0.3	4.4	21.3
Lung	21.5	38.3	1.6	31.0	76.7
Lymph notes	25.5	33.9	0.2	2.1	45.4
Muscle	100.0	19.5	1.2	1.5	25.6
*Pancreas*	32.6	21.5	3.7	25.7	12.9

The expression profiles of the five genes in 11 porcine tissues from four animals (crossbreeds between Landrace, Yorkshire and Duroc). The expression levels are normalised to 3 internal reference genes and scaled to 100 for the tissue with highest expression. The levels can be compared between tissues but not between genes. Expression levels in the four intestinal tissues are in bold.

**Figure 1:**
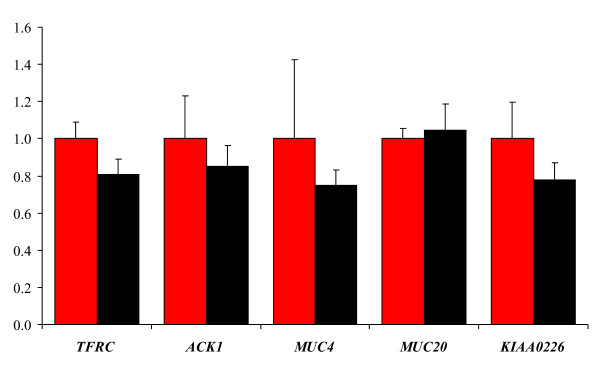
**Expression of the porcine *TFRC, ACK1, MUC4, MUC20 *and *KIAA0226 *genes**. The expression levels in intestinal cells from five susceptible Yorkshire animals (red) and five resistant Yorkshire animals (black). The expression levels are normalised to 3 internal reference genes and scaled, and the expressions levels can therefore not be compared between genes. Error bars are shown as standard error of mean.

The expression levels of the five genes in enterocytes from *jejunum *from a total of 10 Yorkshire animals phenotyped as either susceptible or resistant to F4ab/ac infection (five of each) were measured by qPCR. ANOVA test of the qPCR data did not reveal any significant differential expression of the five candidate genes between susceptible and resistant animals (Figure 1).

## Discussion

**Figure 2:**
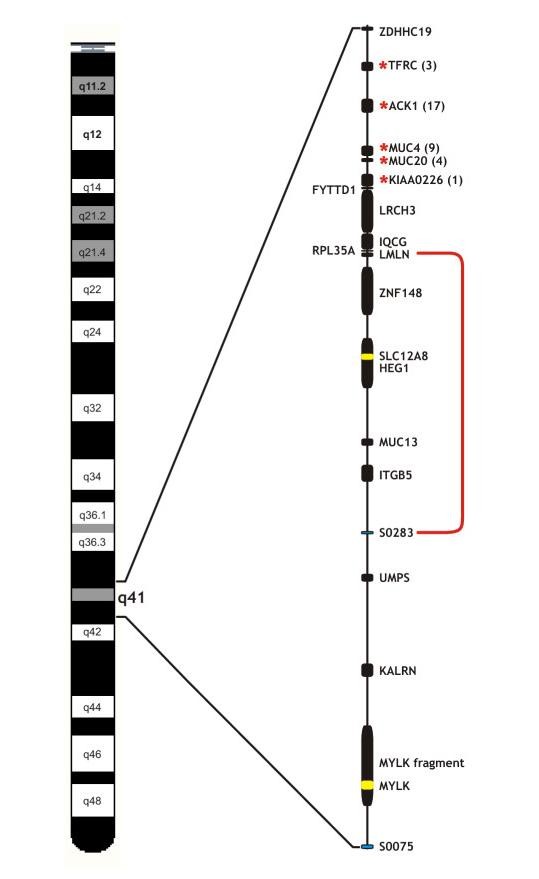
**Localisation of the F4ab/ac candidate region on SSC13**. The gene annotations, order and approximate scale are deduced from the porcine map in Ensembl (Sscrofa9). The localisation of the five investigated genes; *TFRC *(100.8 Mb), *ACK1 *(100.9 Mb), *MUC4 *(101.0 Mb), *MUC20 *(101.1 Mb) and *KIAA0226 *(101.1 Mb) are marked by a red *, and the number of identified polymorphisms in each gene is indicated in brackets. The newly reduced region from the *LMLN *(101.4 Mb) gene to microsatellite S0283 (102.0 Mb) is enclosed by the red line. This figure is modified from previously published figures [6,25]

Infection by enterotoxigenic *Escherichia coli *(ETEC) F4ab/ac is a major cause of death in piglets and identification of the causative gene(s) will have an impact on breeding programs. By selecting for resistant animals the cost for the pig production could be reduced and livestock welfare would be increased. Susceptibility to this infection is inherited as a Mendelian dominant trait, and we characterised the porcine *MUC4 *gene and the four neighbouring genes for exonic and intronic mutations. The localisation of these investigated five genes on pig chromosome 13 is depicted in figure 2. A total of six polymorphisms matched perfectly the ETEC F4ab/ac genotype in four animals of the Large White breed and Wild Boars, and these six polymorphisms were genotyped in 42 additional animals of different breeds. Two of these, polymorphisms 3 and 34, were shown to be significantly associated with the F4ab/ac phenotype with chi-squares of 26.2 and 31.7, respectively. These two polymorphisms are located in either an exon or an intron-sequence in close proximity to an exon. One polymorphism in intron 11 of *TFRC *(No. 3) is located 61 bases upstream of exon 12, and one CTG-TTG synonymous substitution is located in the last exon of *KIAA0226 *(No. 34). These polymorphisms are not obvious causative mutations for ETEC F4ab/ac susceptibility, but they are both predicted to either introduce or disrupt a splice regulatory sequence using Human Splicing Finder V2.4 [18]. The cDNA sequence for *TFRC *has been investigated previously using both animals resistant and susceptible to ETEC infection [13,14] but no evidence of splice variants were reported. The intron 11 polymorphism is thus not likely to influence the regulation of splicing of the primary messenger RNA. As for the *KIAA0226 *gene, we have compared sequences from genomic and cDNA reads and have not found evidence of either introduction/or skipping of exons in relation to the ETEC F4ab/ac status. However it can not be excluded that one of these two polymorphisms are located in a regulatory region, necessary for proper translation, thereby affecting the level of protein synthesis.

An LD test has revealed a D' value of 0.94 between polymorphisms 3 and 34, indicating a tight association, where the polymorphism in *KIAA0226 *shows the highest degree of association with the ETEC F4ab/ac phenotype (q-square value of 31.7). Several studies using various pig breeds have indentified markers in the candidate region that are tightly associated, suggesting that the whole region between and including the *TFRC *and *MUC13 *genes is in tight linkage disequilibrium with the ETEC F4ab/ac locus [5,10,14,21,22]. However, the polymorphism in intron 7 of *MUC4*, which is in complete LD with the ETEC F4ab/ac locus in both a Swiss and Swedish porcine population [5], is only associated and not in complete LD with the F4ab/ac locus in a Belgian breed [23]. A similar conclusion was reached in a Chinese study [24]. These discrepancies are due to a high degree of LD in the candidate region, the use of different breeds and populations and uncertainties in relation to the phenotyping methods.

Recently, a recombination event was observed in a Swiss boar, which reduces the candidate region considerably. This region now maps from *LMLN *to S0283 (Figure 2), distal to *KIAA0226*, thereby excluding the five candidate genes analysed here from the candidate region [25]. However these data are all based on a small population and a more comprehensive study of this recombination event is needed for clarification.

Since animals susceptible to ETEC infection express the F4ab/ac receptor irrespective of the presence of the bacteria and since susceptibility might be reflected at the level of gene transcription, we investigated the transcriptional profiles of the five candidate genes. We constructed an expression panel consisting of various intestinal tissues, muscle and other organs in order to compare the expression patterns across tissues. All five genes were expressed in the intestinal tissues, although the expression of *MUC4 *was very low in all tissues tested when compared to the expression in colon. No significant differences were found between the expression levels in F4ab/ac phenotyped resistant and susceptible animals. This confirms an earlier report on expression of the *MUC20 *gene in pigs infected with ETEC F4ac [26].

A comprehensive cDNA analysis has been performed on *ACK1, MUC4 *and *KIAA0226*. Two transcript variants are known for human *ACK1*. The most highly expressed transcript variant 1 [GenBank: NM_005781] encompass all exons except for exons 2 and 13. Transcript variant 2 [GenBank: NM_001010938] contains an alternative 5'UTR with a start codon in exon 2, and it does not contain exon 15. We identified exon 2 of *ACK1 *using RACE, which suggests the presence of the rarer transcript variant 2 in the pig. However, we failed to identify exon 13 and no published ESTs seem to exist for this exon. We can thus not conclude if pigs contain two transcripts of *ACK1 *similar to humans or if porcine *ACK1 *is different from any of the human transcripts.

Several variants of human *MUC4 *have been reported from mainly tumour tissues and transcript variants 1, 4 and 5 are commonly observed. Transcript variant 1 [GenBank: NM_018406] encompass the full-length transcript, transcript variant 4 [GenBank: NM_004532] lacks exon 2, which contains a large variable tandem repeat region. Transcript variant 5 [GenBank: NM_138297] lacks both exons 2 and 3. Using primers located in porcine *MUC4 *exon 1 and exon 5-6, it was possible to amplify a specific product from cDNA containing exons 1, 3-5 (data not shown). This suggests the presence of a porcine transcript variant, which is dissimilar to human transcript variants 1 and 5, and possibly similar to human transcript variant 4. By extending the elongation time during the PCR cycle (1 min. to 6 min.), it was also possible to obtain several products differing approximately 450 nt. in size, visualised as ladder bands on an agarose gel. Sequencing these products revealed the presence of one to four repeats of 456 nt., which suggests that pigs also express an orthologue of human *MUC4 *transcript variant 1.

Two transcript variants for the human *KIAA0226 *gene have been reported. The longest transcript variant 1 [GenBank: 001145642] lacks exon 2, and transcript variant 2 contains an alternative first exon (exon 2) and lacks exon 9. The whole coding region of porcine *KIAA0226 *was amplified from cDNA and there was no evidence of the presence of exon 9, suggesting that the porcine *KIAA0226 *transcript is similar to human transcript variant 2.

These studies of the coding regions of the three genes were all performed on cDNA generated from total RNA purified from *jejunum *tissue. The absent transcript variants could therefore still be present in others tissues, and to exclusively confirm the number of transcript variants of these genes, a northern blot is needed, and preferably from several different porcine tissues. In this study the overall expression levels of the five genes have been measured by QPCR using primers located in exons, which are present in all of the known transcript variants. This approach, however, introduces the bias that differences in gene expression between two transcript variants will not be detected.

## Conclusion

Gene characterisation of the porcine orthologues of human genes; *TFRC, ACK1, MUC4, MUC20 *and *KIAA0226 *contributes to both the assembly and annotation of the porcine genome, and is beneficial to future studies of these genes in pig. Almost all coding parts of the candidate genes have been investigated for mutations. Although none of the identified polymorphisms are obvious causative candidate mutations, we cannot exclude that these genes are *bona fide *candidates for susceptibility to ETEC F4ab/ac infection. We have not detected any differences in expression levels of the tested candidate genes between resistant and susceptible animals, but we cannot exclude that alternative splice forms exist or that post translational mechanisms contribute to disease susceptibility.

## Competing interests

The authors declare that they have no competing interests.

## Authors' contributions

MJJ has been involved in all aspects of planning and performing this study, including drafting this manuscript. SC performed the qPCR, and participated in the project coordination together with CB, LF, MF and CBJ. DJ, IE, PV, SN, HUB and AR are responsible for phenotyping of the animals and GE and SSK were involved in the gene characterisation. All authors read and approved the final manuscript.

## Supplementary Material

Additional file 1 **Complete list of primer sequences, amplicon sizes and amplicon coverage**. Click here for file
